# Effect of Prebiotic Dietary Supplement Acacia senegal on Hormonal and Metabolic Markers in Polycystic Ovary Syndrome Patients: A Pilot Study

**DOI:** 10.7759/cureus.45480

**Published:** 2023-09-18

**Authors:** Reem I Mohamed, Ibrahim M Daoud, Awadia G Suliman, Lamis Kaddam

**Affiliations:** 1 Department of Physiology, Faculty of Medicine, Alneelain University, Khartoum, SDN; 2 Department of Obstetrics and Gynaecology, Faculty of Medicine, Alneelain University, Khartoum, SDN; 3 Faculty of Radiological Sciences and Medical Imaging, Alzaiem Alazhari University, Khartoum, SDN; 4 Department of Diagnostic Radiology Technology, College of Applied Medical Sciences, Taibah University, Almadinah Almunawarah, SAU; 5 Department of Physiology, Faculty of Medicine, Alneelain University, Khartoum, Sudan, SDN; 6 Department of Physiology, King Abdulaziz University Faculty of Medicine, Rabigh, SAU

**Keywords:** lh/fsh ratio, gum arabic (ga), lh, fsh, polycystic ovary syndrome (pcos), cholesterol, insulin, testosteron, metabolic markers, hormonal profiles

## Abstract

Background

The most prevalent endocrine condition affecting women of reproductive age is polycystic ovarian syndrome (PCOS), which is linked to a variety of metabolic abnormalities. Although the pathogenesis of PCOS is not fully understood, it is known that oxidative stress, altered gut microbiome, and increased gonadotrophin-releasing hormone play a significant role. Gum arabic (GA) is an edible, dried, gummy exudate from the *Acacia senegal *tree, well-known for its prebiotic and antioxidant effects. The main objective of the study was to assess the changes in hormonal and metabolic profiles in PCOS patients after the ingestion of gum arabic.

Method

This was a clinical trial conducted on fifteen patients suffering from PCOS, with a mean age of 27.8 years (20-39 years). All patients experienced irregular cycles. Hormonal and metabolic markers (follicular stimulating hormone (FSH), luteinizing hormone (LH), total testosterone (TT), fasting insulin, total cholesterol (TC), and glycosylated hemoglobin (HBA1c) were measured before and after the ingestion of gum arabic (30 g/day of GA dissolved in 250 ml water for eight weeks) on the second day of the menstrual cycle after granting ethical approval from the National Medicine and Poisons Board and from the participants of the study.

Results

The study demonstrated a significant decrease in the luteinizing hormone level, FSH/LH ratio, and cholesterol pre- and post-gum arabic ingestion (p-values 0.001, 0.013, and 0.007, respectively). Follicular stimulating hormone slightly reduced post-ingestion with no significant difference (p-value 0.414). No significant changes were seen in the testosterone, insulin, or HBA1c levels.

Conclusion

The study concluded that gum arabic ingestion for eight weeks decreases the luteinizing hormone and LH/FSH ratio and improves the metabolic profile by reducing the cholesterol level in PCOS patients.

## Introduction

Polycystic ovarian syndrome (PCOS) is the most frequent endocrine disorder in reproductive females around the world. Depending on the diagnostic criteria used, the prevalence is 5%-20%. According to specialist society recommendations, the presence of at least two of the following three criteria is required for the diagnosis of PCOS: persistent anovulation, hyperandrogenism, and polycystic ovaries. Infertility, metabolic syndrome, obesity, impaired glucose tolerance, type 2 diabetes mellitus (DM-2), cardiovascular risk, and endometrial cancer all are associated with PCOS [1,2].

PCOS etiology has been linked to altered luteinizing hormone (LH) function, insulin resistance (IR), and a probable proclivity for hyperandrogenism. According to one explanation, the underlying insulin resistance exacerbates hyperandrogenism by decreasing sex hormone-binding globulin production and promoting adrenal and ovarian androgen synthesis, hence increasing androgen levels. These androgens cause irregular menstruation and physical signs of hyperandrogenism [2].

Women with PCOS are managed differently depending on their symptoms. These could include infertility caused by ovulatory failure, menstrual problems, or androgen-related symptoms [3].

PCOS-related hyperandrogenism and central obesity can have a significant impact on a woman's fertility and overall health. Weight loss has been shown to improve hormonal imbalances and increase the likelihood of ovulation and pregnancy in women with PCOS. Even a modest weight loss of 5% of the initial weight can have positive effects. The treatment of obesity in PCOS often involves lifestyle modifications such as changes in diet and exercise. Medical and surgical options may also be considered. It is important to note that weight loss is generally recommended for women with a BMI > 25-27 kg/m2 [3]. For anovulatory infertility, medications like clomiphene citrate (CC), metformin, and insulin-sensitizing drugs can be used to induce ovulation. In cases where endometrial proliferation needs to be controlled, cyclic progestin or oral contraceptives with a combination of estrogen and progestin may be prescribed. Androgen-related symptoms in PCOS can be managed with various treatment options. These can include oral contraceptives, estrogen-progestin combination therapy, antiandrogens, gonadotropin-releasing hormone agonists, and insulin-lowering agents. In addition to conventional medical treatments, alternative options such as kinesiology, herbalism, and acupuncture may also be considered [3].

Gum arabic (GA) is a soluble fiber derived from the *Acacia senegal *and *Acacia seyal *trees in Sub-Saharan Africa, particularly Sudan. It is a significant medicinal herb used in alternative medicine and has been deemed suitable for daily consumption since 1969 [4]. GA is indigestible to humans and animals and ferments in the colon to produce short-chain fatty acids, offering potential health advantages such as prebiotic effects, lower plasma cholesterol levels, anticarcinogenic activity, and antioxidant activity [5-8]. Epidemiological studies have shown that high dietary fiber intake is associated with improved fat metabolism, satiation, fullness, glycemic index, stomach emptying, and gut hormone production [9,10]. Additionally, GA reduces intestinal glucose absorption through interaction with intestinal Na+-coupled glucose transporter (SGLT1) in mice, and during a high-fat meal, GA substantially reduces body weight, fasting plasma glucose, and fasting insulin concentrations [11,12].

Several studies have been conducted in Sudan in humans to evaluate the medicinal herbal effect of GA, such as in reducing BMI and body fat in healthy females, decreasing oxidative stress and inflammatory markers in hemodialysis patients, improving poor glycemic control, and improving lipid and BMI in diabetic type II patients, lowering total cholesterol in hyperlipidemia patients, and used to increased fetal hemoglobin in sickle cell anemia (SCA) patients [11,13,14,15,16]

PCOS is associated with obesity, impaired glucose tolerance, and lipids disorders. Gum arabic is a herbal intake proven to cause a reduction in BMI and lower plasma cholesterol, fasting glucose, and insulin level, so it was used as an alternative medicinal herb in PCOS females. To our knowledge, there are no previous studies conducted to explore the effect of gum arabic ingestion among polycystic ovary syndrome (PCOS) patients. Therefore, this study adds valuable information for both doctors and patients to expand their options in PCOS management.

## Materials and methods

A pilot study was conducted in Bannon Fertility Center in Sudan, Khartoum in the period from January to August 2021, to explore the effect of gum arabic ingestion on the metabolic and hormonal profile of polycystic ovary syndrome (PCOS) patients. Fifteen patients diagnosed with PCOS based on Rotterdam Criteria (2003) [17], non-diabetic and normotensive suffering from primary or secondary infertility and not taken any treatment for PCOS and infertility before gum arabic ingestion were recruited, after being granted ethical approval from the Institutional Review Board (IRB) and by Clinical Trials on Human and Animals Committee, National Medicines and Poisons Board, Federal Ministry of Health (approval no.0446749/NMPB). Females with thyroid disorders or who refused to participate in the study were excluded.

Two classes of data were collected, one before ingestion of gum arabic (GA) and another 8 weeks after ingestion of gum arabic (GA), Gum Arabic Company in Khartoum, Sudan provided GA in powder form. The powder is a pure extract mechanically produced from the wild *Acacia senegal *tree with no additives. Based on previous studies, the GA administration was used for 8 weeks, with a daily dose of 30 grams [13,16]. GA was packed as 15 g/sachet. The patients were instructed to dissolve two sachets in water and take them early in the morning on an empty stomach. Participants received 60 packs per month for eight weeks.

Hormonal profiles were obtained for PCOS women with regular and irregular menstrual cycles on the second day of the menstrual cycle, which is the standard for the basic level of hormones (early proliferative phase). A 10 ml blood sample was taken twice, before and after the gum arabic ingestion for 8 weeks. For women with irregular menstrual cycle, the investigators waited for the onset of the menstrual cycle and took the blood sample on the second day prior to ingesting GA, and then also after taking the GA for 8 weeks waited for the onset of first menstruation post-ingestion and took the second sample. Blood samples were centrifuged for 10 min at 4000 rpm to separate the serum. The extracted serum was divided into three sample aliquots and frozen immediately at -70°C until assay. Laboratory tests were performed after overnight fasting (8-10 hours). The levels of follicular stimulating hormones (FSH), luteinizing hormones (LH), testosterone, and insulin were evaluated using Immunoenzymometric Assay TOSOH AIA-1800 Chemistry Analyzer (Tosoh Bioscience, South San Francisco, USA), total cholesterol was determined by Auto-Chemistry Analyzer CS-T180 (Dirui Medical Technology, Jilin, China) and HBA1c was done by analyzer Finecare (Guangzhou Wondfo Biotech, Guangzhou, China).

Data were entered in Excel sheet Microsoft 10 (Microsoft Corporation, Redmond, USA), and were analyzed using SPSS version 23 (IBM Corp., Armonk, USA). Quantitative data were expressed as mean and standard deviation, and the paired sample t-test was used to compare the results before and after administration of gum arabic powder. A p-value ≤0.05 was considered statistically significant.

## Results

**Figure 1:**
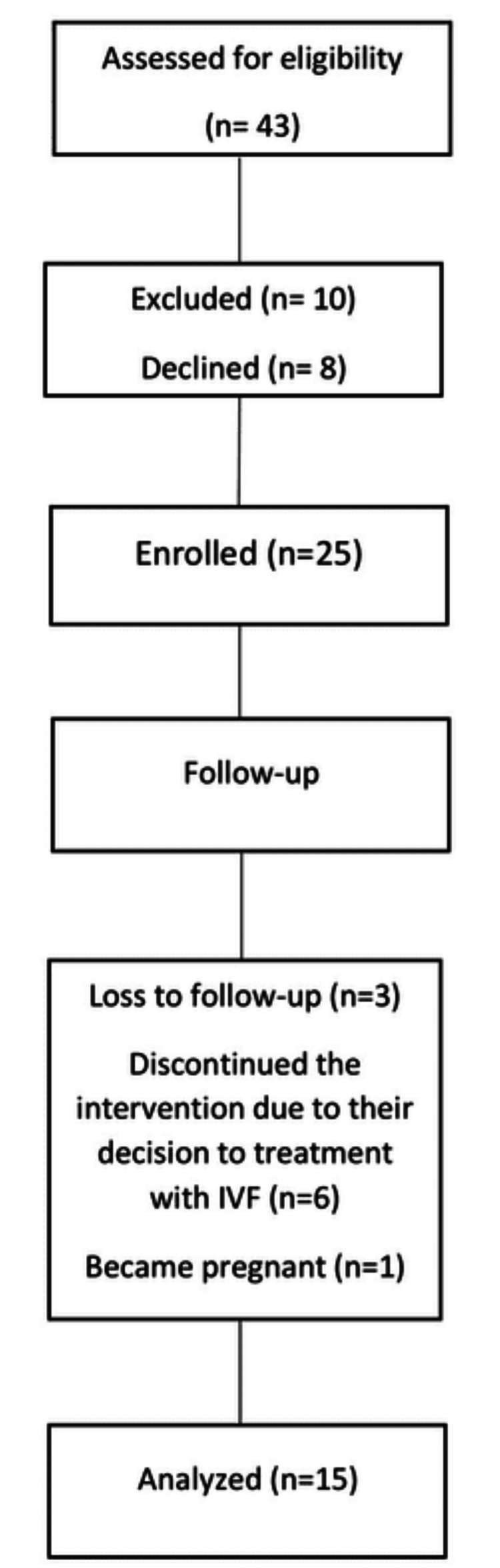
The chart shows the number of patients included in the study. IVF: in vitro fertilization

We assessed 43 patients for eligibility, of them 10 were excluded, eight declined, 25 enrolled in the study, 10 lost during the intervention (one became pregnant, six chose in vitro fertilization (IVF) as a method of treatment, and three lost in follow-up). A total of 15 patients were included in the final analysis (Figure 1).

The study participants' ages ranged from 20 to 39 years, with a mean age of 27.80±5.64 years. More than half of them were in the age group 20-29 years, and the majority of them (80%) were housewives, with (33.3%) suffering from hirsutism (Table 1).

**Table 1:** Demographic characteristics and complaints

Variables	Frequency	Percentage
Age group		
20-29	8	53.3
30-39	7	46.7
Mean (27.80±5.64 years)		
Occupation		
Housewife	12	80
Employee	3	20
Hirsutism		
Yes	5	33.3
No	10	66.7

This study clarified that the 8 weeks of oral intake of gum arabic (GA) significantly decreases the level of LH hormones and the LH/FSH ratio. The mean LH, LH/FSH ratio, and cholesterol, were 10.94±6.67 mlU/L, 1.60±0.94, and 162.56±62.20 mlU/L, respectively, before and 5.03±3.60 mlU/L, 0.99±0.83, and 127.90±25.92 mlU/L, respectively, after ingestion of gum arabic, p-value < 0.05, while no significant change was seen in FSH, testosterone, HbA1c, and insulin, p-value > 0.05 (Table 2).

**Table 2:** The mean hormonal and laboratory profiles in PCOS patients pre and post-ingestion of GA (Gum Arabic) FSH: follicle-stimulating hormone; LH: luteinizing hormone; HbA1c: glycated hemoglobin

Hormonal and metabolic markers (pre- and post-intervention)	Mean	SD	P-value
FSH (mIU/L) Pre-ingestion	6.90	2.45	0.414
FSH (mIU/L) Post-ingestion	6.18	2.30	
LH ((mIU/L) Pre-ingestion	10.94	6.76	0.001
LH (mIU/L) Post-ingestion	5.03	3.60	
LH/FSH Pre-ingestion	1.60	0.94	0.013
LH/FSH Post-ingestion	0.99	0.83	
Testosterone Pre-ingestion	0.33	0.08	0.912
Testosterone Post-ingestion	0.34	0.14	
Cholesterol (mg/ml) Pre-ingestion	162.56	62.20	0.007
Cholesterol (mg/ml) Post-ingestion	127.90	25.92	
Insulin Pre-ingestion	22.48	20.96	0.555
Insulin Post-ingestion	18.82	13.47	
HbA1c Pre-ingestion	5.71	0.89	0.579
HbA1c Post-ingestion	5.61	0.52	

## Discussion

Polycystic ovarian syndrome (PCOS) is the most common endocrine disease in women of reproductive age, associated with multiple metabolic abnormalities. Gum arabic (GA) is a comestible, dried, sticky exudate from the *Acacia senegal *tree [9], known for its prebiotic and antioxidant properties. The main objective of this study was to explore the changes in hormonal and metabolic profiles in PCOS patients after gum arabic ingestion for 8 weeks.

In PCOS, there is increased gonadotropin-releasing hormone (GnRH) pulse frequency, which increases the frequency and pulse amplitude of LH over FSH production resulting in a high LH/FSH ratio [18,19], with the association of the high concentrations of LH in the follicular phase ovulation, does not occur [20]. In the present study, GA significantly reduced LH hormone and the LH/FSH ratio and exhibited no significant change regarding FSH level. One of the possible explanations for such results is the restoration of GnRH pulse frequency balance which directly affects LH frequency and amplitude without a significant change in the FSH. The GA could exert this action through its prebiotic properties. The gut microbiomes exert a profound influence on key brain processes through the microbiota-gut-brain axis [21-23]. It directly acts on the hypothalamic-pituitary-adrenal (HPA) axis [24], produces short-chain fatty acids that possess neuroactive properties, and synthetizes a number of neurotransmitters [25-28]. One of those neurotransmitters is dopamine (DA). DA is known to be a potent inhibitor of GnRH neuron excitability [29-31]. Many PCOS-associated morbidities are noticed to be related to the high LH/FSH ratio thus improving the ratio is supposed to enhance the metabolic profile of the patients.

Abnormal lipid metabolism can contribute to the pathophysiology of hyperandrogenism, IR, oxidative stress, and infertility in PCOS. Lipid abnormalities are common in women with polycystic ovary syndrome (PCOS), with mild hypercholesterolemia being common. Obesity affects oocyte quality and early embryo growth, which is triggered by lipo-toxicity-induced endoplasmic reticulum stress, mitochondrial dysfunction, and apoptosis. Studies have shown that androgens play a significant role in hyperlipidemia, while hypomethylated genes related to lipid synthesis may promote the synthesis of steroid hormones including androgen. Hypercholesterolemia is also associated with oxidative stress, as studies have shown that hypercholesterolemia reduces liver sirtuin 1 (SIRT1) and induces hepatic oxidative stress. PCOS-induced rats model that was on a high-fat diet showed ovarian changes suggesting infertility is associated with dyslipidemia in PCOS [32]. In this study, the cholesterol level decreased significantly after ingestion of GA, and this result is consistent with the findings of other experiments [14,15,33]. Dietary fibers such as GA can lower the cholesterol level by many mechanisms, including the viscosity effect of dietary fibers, the increase in fecal bile acids, alteration of lipid metabolism, and an increase in the number of lipoprotein receptors in the body [34-36].

GA proved to cause a decrease in fasting insulin and glucose [11-12]. Our study revealed a mild insignificant reduction probably because more than half of our patients had a normal baseline insulin level. A study done in mice in 2010 confirmed that Indeed the hyperglycemic effect of excessive glucose intake was significantly blunted by GA treatment [12]. Moreover, GA treatment prevented body weight gain following a four-week treatment with a glucose-rich diet despite similar fluid and food intake in untreated and GA-treated mice [12]. PCOS women with IR had higher frequencies of menstrual irregularity, hyperandrogenism, infertility or subfertility and obesity [37], Homeostasis mode assessment estimated insulin resistance (HOMA-IR ) more accurate than fasting insulin for evaluating PCOS women [37], so a further assessment of the effect of GA on the insulin status of PCOs patients is needed. Studies conducted in type 2 diabetic patients found that the administration of GA for 3 months caused a significant decrease in HbA1c, BMI, and lipid accumulation product and an insignificant decrease in triglycerides [14,38]. In this study, the ingestion of GA for 8 weeks caused a decrease in HbA1c, but it was insignificant (p-value > 0.05); this could be explained by the differences in GA administration duration.

In spite of the impact of gum arabic (GA) ingestion for 8 weeks on hormonal and metabolic markers in PCOS patients that were found in this study, it is important to note that the study has several limitations. One of the main limitations is the small sample size which limits the generalizability and reliability of the study findings. The reluctance of some patients towards herbal therapy, particularly those seeking assisted reproductive therapies (the study was conducted in a specialized private center and most of the patients aimed to do IVF) influenced the recruitment and participation of PCOS patients and contributed to the smaller sample size. Another limitation is the absence of a placebo group. So it is crucial to conduct further research with a larger sample size to improve the statistical power and enhance the generalizability of the findings. It will also enable researchers and clinicians to better understand the potential benefits and effects of GA as an alternative herbal medicinal option for managing PCOS.

## Conclusions

The study concluded that the prebiotic dietary supplementation (*Acacia senegal*/gum arabic) for 8 weeks in a patient with PCOS (polycystic ovarian syndrome) reduced luteinizing hormone and LH/FSH ratio and improved the metabolic profile by lowering cholesterol levels.

## Appendices

The study was presented earlier as a proceeding abstract in Euro-physiology in 2022.

## References

[1] Azziz R, Carmina E, Chen Z (2016). Polycystic ovary syndrome. Nat Rev Dis Primers.

[2] Williams T, Mortada R, Porter S (2016). Diagnosis and treatment of polycystic ovary syndrome. Am Fam Physician.

[3] Badawy A, Elnashar A (2011). Treatment options for polycystic ovary syndrome. Int J Womens Health.

[4] Ahmed AA (2018). Health benefits of gum arabic and medical use. Gum Arabic: Structure, Properties, Application and Economics.

[5] Phillips GO, Ogasawara T, Ushida K (2008). The regulatory and scientific approach to defining Gum Arabic (Acacia senegal and Acacia seyal) as a dietary fibre. Food Hydrocoll.

[6] Sharma RD (1985). Hypocholesterolemic effect of gum acacia in men. Nutr Res.

[7] Nasir O, Wang K, Föller M (2010). Downregulation of angiogenin transcript levels and inhibition of colonic carcinoma by gum arabic (Acacia senegal). Nutr Cancer.

[8] Ali BH, Al-Qarawi AA, Haroun EM, Mousa HM (2003). The effect of treatment with gum Arabic on gentamicin nephrotoxicity in rats: a preliminary study. Ren Fail.

[9] Ali BH, Ziada A, Blunden G (2009). Biological effects of gum arabic: a review of some recent research. Food Chem Toxicol.

[10] Chandalia M, Garg A, Lutjohann D, von Bergmann K, Grundy SM, Brinkley LJ (2000). Beneficial effects of high dietary fiber intake in patients with type 2 diabetes mellitus. N Engl J Med.

[11] Babiker R, Merghani TH, Elmusharaf K, Badi RM, Lang F, Saeed AM (2012). Effects of Gum Arabic ingestion on body mass index and body fat percentage in healthy adult females: two-arm randomized, placebo controlled, double-blind trial. Nutr J.

[12] Nasir O, Artunc F, Wang K (2010). Downregulation of mouse intestinal Na(+)-coupled glucose transporter SGLT1 by gum arabic (Acacia Senegal). Cell Physiol Biochem.

[13] Ali NE, Kaddam LA, Alkarib SY (2020). Gum Arabic (Acacia Senegal) augmented total antioxidant capacity and reduced C-reactive protein among haemodialysis patients in phase ii trial. Int J Nephrol.

[14] Babiker R, Elmusharaf K, Keogh MB, Banaga ASI (2017). Metabolic effect of Gum Arabic (Acacia Senegal) in patients with Type 2 Diabetes Mellitus (T2DM): Randomized, placebo controlled double blind trial. Functional Foods Health Dis.

[15] Mohamed RE, Gadour MO, Adam I (2015). The lowering effect of Gum Arabic on hyperlipidemia in Sudanese patients. Front Physiol.

[16] Kaddam L, FdleAlmula I, Eisawi OA, Abdelrazig HA, Elnimeiri M, Lang F, Saeed AM (2015). Gum Arabic as fetal hemoglobin inducing agent in sickle cell anemia; in vivo study. BMC Hematol.

[17] (2004). Revised 2003 consensus on diagnostic criteria and long-term health risks related to polycystic ovary syndrome (PCOS). Hum Reprod.

[18] Yen SS (1980). The polycystic ovary syndrome. Clin Endocrinol (Oxf).

[19] McKenna TJ (1988). Pathogenesis and treatment of polycystic ovary syndrome. N Engl J Med.

[20] Homburg R (1996). Polycystic ovary syndrome - from gynaecological curiosity to multisystem endocrinopathy. Hum Reprod.

[21] Erny D, Hrabě de Angelis AL, Jaitin D (2015). Host microbiota constantly control maturation and function of microglia in the CNS. Nat Neurosci.

[22] van de Wouw M, Boehme M, Lyte JM (2018). Short-chain fatty acids: microbial metabolites that alleviate stress-induced brain-gut axis alterations. J Physiol.

[23] Borre YE, O'Keeffe GW, Clarke G, Stanton C, Dinan TG, Cryan JF (2014). Microbiota and neurodevelopmental windows: implications for brain disorders. Trends Mol Med.

[24] Sudo N, Chida Y, Aiba Y (2004). Postnatal microbial colonization programs the hypothalamic-pituitary-adrenal system for stress response in mice. J Physiol.

[25] Fung TC, Olson CA, Hsiao EY (2017). Interactions between the microbiota, immune and nervous systems in health and disease. Nat Neurosci.

[26] Dalile B, Van Oudenhove L, Vervliet B, Verbeke K (2019). The role of short-chain fatty acids in microbiota-gut-brain communication. Nat Rev Gastroenterol Hepatol.

[27] Sherwin E, Dinan TG, Cryan JF (2018). Recent developments in understanding the role of the gut microbiota in brain health and disease. Ann N Y Acad Sci.

[28] González-Arancibia C, Urrutia-Piñones J, Illanes-González J, Martinez-Pinto J, Sotomayor-Zárate R, Julio-Pieper M, Bravo JA (2019). Do your gut microbes affect your brain dopamine?. Psychopharmacology (Berl).

[29] Henderson HL, Townsend J, Tortonese DJ (2004). Direct effects of prolactin and dopamine on the gonadotroph response to GnRH. J Endocrinol.

[30] Liu X, Herbison AE (2013). Dopamine regulation of gonadotropin-releasing hormone neuron excitability in male and female mice. Endocrinology.

[31] Beydoun HA, Beydoun MA, Wiggins N, Stadtmauer L (2012). Relationship of obesity-related disturbances with LH/FSH ratio among post-menopausal women in the United States. Maturitas.

[32] Liu Q, Xie YJ, Qu LH, Zhang MX, Mo ZC (2019). Dyslipidemia involvement in the development of polycystic ovary syndrome. Taiwan J Obstet Gynecol.

[33] Mee KA, Gee D (1997). Apple fiber and gum arabic lowers total and low density lipoprotien cholestrol levels in men with mild hypercholesrtolemia. J Acad Nutr Dietet.

[34] Marciani L, Gowland PA, Spiller RC (2000). Gastric response to increased meal viscosity assessed by echo-planar magnetic resonance imaging in humans. J Nutr.

[35] Moundras C, Behr SR, Rémésy C, Demigné C (1997). Fecal losses of sterols and bile acids induced by feeding rats guar gum are due to greater pool size and liver bile acid secretion. J Nutr.

[36] Fernandez ML, Ruiz LR, Conde AK, Sun DM, Erickson SK, Mcnamara DJ (1995). Psyllium reduces plasma LDL in Guinea-pigs by alterting hepatic cholesterol homeostasis. J Lipid Res.

[37] Majid H, Masood Q, Khan AH (2017). Homeostatic Model Assessment for Insulin Resistance (HOMA-IR): a better marker for evaluating insulin resistance than fasting insulin in women with polycystic ovarian syndrome. J Coll Physicians Surg Pak.

[38] Babiker R, Elmusharaf K, Keogh MB, Saeed AM (2018). Effect of Gum Arabic (Acacia Senegal) supplementation on visceral adiposity index (VAI) and blood pressure in patients with type 2 diabetes mellitus as indicators of cardiovascular disease (CVD): a randomized and placebo-controlled clinical trial. Lipids Health Dis.

